# Supplementary material for: Natural forgetting reversibly modulates engram expression
Source: eLife. 2024 Nov 5;12:RP92860. doi: 10.7554/eLife.92860 (PMC11537488; doi:10.7554/eLife.92860)
Supplement: Supplementary file 1. — Control group (Cont) and experimental group (Exp). [file elife-92860-supp1.docx]

**Supplementary Table 1. Parameter Estimates.** Control group (Cont) and Experimental group (Exp).

|  | **Alpha** | **Beta** | **Kappa** |
| --- | --- | --- | --- |
| **Enrichment** | Cont: 0.072; Exp: 0.0 | Cont: -0.695; Exp: -0.48 | Cont: 23.12; Exp: 19.668 |
| **Rac1 inhibition** | Cont: 0.101; Exp: 0.013 | Cont: -0.651; Exp: -0.736 | Cont: 29.209; Exp: 13.841 |
